# Quality improvement project to optimize enteral nutrition in a tertiary hospital's surgical ICU

**DOI:** 10.1186/cc14475

**Published:** 2015-03-16

**Authors:** J Li, LY Koh, JH Yang, C Khoo, T Ter, BH Tan

**Affiliations:** 1 National University Health System, Singapore

## Introduction

Optimizing enteral nutrition early has been shown to be beneficial in critically ill patients. However, underfeeding is still a common problem. The critically ill surgical patient often presents with additional challenges to optimal enteral feeding. The objective of this study was to improve enteral feeding practices in a surgical ICU.

## Methods

The Clinical Practice Improvement Programme is a local quality improvement initiative involving a multidisciplinary team aimed at identifying and improving deficiencies in the process of delivery of care. A team led by an intensivist, consisting of doctors, surgeons, nurses and a pharmacist, was formed to improve enteral feeding practices in a surgical ICU. The quality improvement methodology was employed. An audit was carried out to determine the problem of underfeeding in the unit. Root cause analyses were conducted and team members identified key barriers to optimal feeding and areas for improvement. Protocols were developed to standardize and encourage early enteral feeding as well as to reduce the time feeds are interrupted for patients who were going for surgeries or for various other reasons. Educational interventions were conducted with lectures to physicians and nurses. Visual aids in the form of screensavers at each bedside computer served as reminders to the team to optimize feeding. A subsequent audit was then conducted to determine the improvement in achieving the desired outcomes, namely the amount of calories and proteins received as well as the proportion of patients who achieved >70% of their target calories and proteins. We considered target calories to be 25 kcal/kg/day and target proteins to be 1.5 g/kg/day.

## Results

Patients received more calories (78.3% vs. 59.1%) and more proteins (70.2% vs. 54.6%) post implementation. The mean percentage of patients in the post group who achieved >70% of required calories was 80.1% versus 30.9% in the pre group. The mean percentage of patients who achieved >70% of required proteins was 58.3% versus 32.1% in the pre group.

## Conclusion

The multipronged approach of the quality improvement methodology helped to increase the provision of calories and proteins in our population of critically ill surgical patients. However, there is still room for improvement in terms of achieving optimal enteral nutrition targets early in our population. There is also a need to look into sustaining such results.

